# The Effects of Drought and Re-Watering on Non-Structural Carbohydrates of *Pinus tabulaeformis* Seedlings

**DOI:** 10.3390/biology10040281

**Published:** 2021-03-30

**Authors:** Xinyi Guo, Changhui Peng, Tong Li, Jingjing Huang, Hanxiong Song, Qiuan Zhu, Meng Wang

**Affiliations:** 1 Center for Ecological Forecasting and Global Change, College of Forestry, Northwest A&F University, Yangling 712100, China; guo.xinyi@nwafu.edu.cn (X.G.); litong585072@nwafu.edu.cn (T.L.); hjj_2020@nwafu.edu.cn (J.H.); 2 Department of Biology Sciences, Institute of Environment Sciences, University of Quebec at Montreal, P.O. Box 8888, Station Centre-Ville, Montreal, QC H3C 3P8, Canada; song.hanxiong@courrier.uqam.ca; 3 College of Hydrology and Water Resources, Hohai University, Nanjing 210098, China; zhuq@hhu.edu.cn; 4 Key Laboratory of Geographical Processes and Ecological Security in Changbai Mountains, Ministry of Education, School of Geographical Sciences, Northeast Normal University, Changchun 130024, China; 5 State Environmental Protection Key Laboratory of Wetland Ecology and Vegetation Restoration, Institute for Peat and Mire Research, Northeast Normal University, Changchun 130024, China; 6 Jilin Provincial Key Laboratory for Wetland Ecological Processes and Environmental Change in the Changbai Mountains, Changchun 130024, China

**Keywords:** sucrose, glucose, fructose, soluble sugars, Chinese pine, allocation, starch storage, photosynthesis, biomass, hydraulic failure

## Abstract

**Simple Summary:**

Drought is one of the main drivers resulting in carbon imbalance in terrestrial ecosystems and the mortality of plants. How plants can survive under drought stress is becoming a major focus of interest. Non-structural carbohydrates include sugars and starch that are essential to plant metabolism and their roles in drought stress are thought to be critically important. Our study examined the allocation strategies of non-structural carbohydrates for three-year-old *Pinus tabulaeformis* (Chinese pine) seedlings under drought and subsequent re-watering conditions. Our results indicated that *P. tabulaeformis* seedlings showed strong drought resistance by investing limited non-structural carbohydrates to roots and depleting the starch storage in each organ (leaf, twig, stem, and root) to fuel the needs of plant metabolism and osmotic adjustment. Starch storage was first reconstructed after the drought stress was released. Our findings not only prove the important role of non-structural carbohydrates, especially starch storage, in the survival of *P. tabulaeformis* seedlings under drought condition, but also complement the limited studies on allocation strategies of non-structural carbohydrate after the drought stress is released, and broaden our understanding of the physiological mechanisms of plants in response to drought stress.

**Abstract:**

Intense and frequent drought events strongly affect plant survival. Non-structural carbohydrates (NSCs) are important “buffers” to maintain plant functions under drought conditions. We conducted a drought manipulation experiment using three-year-old *Pinus tabulaeformis* Carr. seedlings. The seedlings were first treated under different drought intensities (i.e., no irrigation, severe, and moderate) for 50 days, and then they were re-watered for 25 days to explore the dynamics of NSCs in the leaves, twigs, stems, and roots. The results showed that the no irrigation and severe drought treatments significantly reduced photosynthetic rate by 93.9% and 32.6% for 30 days, respectively, leading to the depletion of the starch storage for hydraulic repair, osmotic adjustment, and plant metabolism. The seedlings under moderate drought condition also exhibited starch storage consumption in leaves and twigs. After re-watering, the reduced photosynthetic rate recovered to the control level within five days in the severe drought group but showed no sign of recovery in the no irrigation group. The seedlings under the severe and moderate drought conditions tended to invest newly fixed C to starch storage and hydraulic repair instead of growth due to the “drought legacy effect”. Our findings suggest the depletion and recovery of starch storage are important strategies for *P. tabulaeformis* seedlings, and they may play key roles in plant resistance and resilience under environmental stress.

## 1. Introduction

Severe and frequent drought events may inhibit forest productivity, leading to the widespread mortality of vegetation and the conversion of forests from carbon (C) sinks to sources [1,2]. Generally, drought-induced shortages of free water in plants constrain the C supply for plant metabolism by inhibiting stomatal conductance and photosynthesis [3]. In addition, severe drought results in xylem cavitation and embolism, and limits the transportation of non-structural carbohydrates (NSCs) and nutrients among different organs [3,4,5]. This results in significant challenges for plants in maintaining C balance and hydraulic transportation under water stress [6,7].

Non-structural carbohydrates, composed of soluble sugars (e.g., glucose, fructose, and sucrose) and starch, are thought to play an important role in the ability of plants to resist environmental stress by acting to buffer the impacts of reductions in carbon balance [8,9]. Different NSC components have different functions [8]. For example, as soluble sugars, glucose and fructose primarily participate in osmotic regulation, and sucrose can be transported to different organs [10,11,12]. Starch is the dominant storage carbohydrate in plants and is converted to soluble sugars under drought stress [8,13]. NSCs are assumed to be an important trait under drought stress because the concentrations of NSC reflect the C balance in woody plants between photosynthetic C assimilation and metabolic C demand [14]. Moreover, the variation of NSC concentrations among different plant organs under drought stress can reflect the allocation strategies of NSCs under the negative C balance [14,15]. 

The relationship between the allocation of NSCs and drought stress has not been sufficiently resolved. Previous studies that have investigated changes in plant NSC concentrations during drought manipulation have shown inconsistent results. For instance, Galvez et al. (2011) found that the NSC concentrations increased two orders of magnitude in aspen seedlings [16], while Adams et al. (2013) found the NSC concentrations decreased in *P. edulis* under drought condition [17]. Gruber et al. (2012) found that there was no impact on NSCs in Scots pine during drought stress [18]. The discrepancies among the previous studies might be attributed to the plant species and also to the different drought intensities and durations [2,17,19]. During the early stage of drought stress, plants need sufficient soluble sugars to participate in xylem embolism refilling to sustain the water flow and phloem turgor pressure to avoid transport failure [20,21]. During prolonged drought, there is growing evidence for NSCs to be allocated to storage under mild and moderate drought conditions, whereas a prolonged severe drought will cause severe hydraulic impairment and complete cessation of photosynthesis, which may completely stop the C allocation and continue consuming the stored NSCs [2,22]. Additionally, most of the current studies have only focused on the responses of NSCs to drought stress in individual plant organs [8,23], which also has likely contributed to the inconsistent results because different organs may have different C allocation strategies. For example, leaf is a source organ that is related to C assimilation [14], while stem and root are important organs for long-term C storage [8,23]. Finally, a systematic understanding of how NSCs contribute to plant survival also requires an investigation regarding the dynamics of NSCs after drought stress is relieved (i.e., re-watering process), which may directly determine the plant’s survival [24]. As demonstrated by Ruehr et al. (2019), after re-watering, though photosynthesis is gradually recovered, the newly assimilated C is insufficient for the plant’s demand during the early stage of re-watering [25]. Therefore, the starch storage will be largely consumed to repair the physiological damage and grow new tissues during the re-watering [25]. In addition, it is still not clear whether different intensities of drought stress have different effects on NSC allocations in seedlings after the drought stress is relieved [2,22]. NSC allocation during the re-watering process may potentially provide new insights regarding the drought resistance and resilience of plants.

*Pinus tabulaeformis* is an endemic tree species and is widely distributed in northwest China, and it is often used for erosion and torrent control projects [26] (p. 251). A previous study has observed that drought can cause stomatal closure and inhibit photosynthesis in *P. tabulaeformis* seedlings [27]. Furthermore, drought may constrain the growth of stems, roots, and leaves [28,29]. NSCs are thought to play an important role in buffering the negative impacts caused by water limitation in *P. tabulaeformis* seedlings. Different allocation strategies among organs and different conversion of stored NSC in various organs during the period of negative C balance caused by drought-related photosynthesis restriction are likely important [30,31]. However, little is known about the mechanisms of NSC allocation among organs of *P. tabulaeformis* under different drought stresses. Concomitantly, the responses of various organs in terms of allocation or depletion of stored carbohydrates to the subsequent re-watering remain to be further investigated. In this study, the effects of three different intensities of drought stress for 50 days followed by re-watering for 25 days (days 51 to 75) on three-year-old *P. tabulaeformis* seedlings were assessed. It was hypothesized that (1) drought would inhibit C assimilation and deplete starch storage to supply the metabolic C demand. This would be manifested by a decreasing starch concentration and an increasing soluble sugar concentration, especially in the roots. After re-watering, it was hypothesized (2) that the newly assimilated C and starch storage in seedlings would be consumed for regrowth, and the starch concentration would significantly decrease, especially under no irrigation and severe drought conditions prior to re-watering. 

## 2. Materials and Methods

### 2.1. Plant Material and Drought Treatments

The study was conducted in the greenhouse of the Northwest Agriculture and Forestry University in Yangling, China. The greenhouse was maintained at the mean daily temperature of 19.5 °C and the humidity of 38.4% over the course of the experiment. The 3-year-old *P. tabulaeformis* seedlings were transplanted in pots (30 cm in diameter and 40 cm in depth; one seedling per pot) on April 2017. The soil is the mixture of local field soil (sieved to remove residual roots and gravel) and sand in a ratio of 5:1. The seedlings were grown under natural light and fertilized with a one-quarter strength solution of Hoagland’s solution prior to the treatments. Before the drought manipulation, all of the seedlings were irrigated regularly to maintain a field water capacity. A randomized block design with four blocks was utilized in our experiment. Before the treatment started, a total of 68 seedlings were randomly assigned to four replicate blocks, and each block contained the control (field capacity) and three drought treatments (no irrigation (<5% field capacity), severe drought (15–20% field capacity), and moderate drought (40–50% field capacity)). The first sampling with 1 seedling sampled from each block (4 in total) was carried out on the day before the treatments started, which represents the initial status of the seedlings. The treatment was started on 28 November 2017. The soil water content was monitored twice a day using reflectometer probes (ML3 ThetaProbe Soil Moisture Sensor, Delta-T Devices Ltd., Burwell, Cambridge, UK). The re-watering began after 50 days of drought manipulation when the seedlings were irrigated regularly to resume the soil water content at field capacity. The re-watering process was maintained for 25 days. Soil moisture contents are shown in Table S1. There was no cold acclimation or dormancy phase that happened in our experiment over the course of the experiment. 

Over the course of this experiment, one seedling from each treatment and each block (n = 4) was sampled for the five different periods in total. Sampling was conducted on the same day, right before the start of drought manipulation (i.e., ‘initial’), 30 days, 50 days, 55 days (5 days after re-watering), and 75 days (25 days after re-watering) after treatments.

### 2.2. The Soil Water Content and Physiological Measurements

The soil sampled from each pot was sieved and air-dried at 105 °C for 48 h to a constant weight to determine the gravimetric soil water content. The net photosynthetic rate, stomatal conductance, and transpiration rate were measured between 9:00 to 11:00 in the morning using a portable photosynthesis analyzer (LI6400, Li-Cor, Lincoln, NE, USA). Mature leaves were selected for the measurement under an active photon flux intensity of 1500 μmol/m^2^·s and an ambient CO_2_ concentration of 450 ± 30 μmol/mol in a red-blue light chamber. Finally, the whole seedlings were sampled destructively and separated into four parts (leaves, twigs, stems, and roots) and oven-dried at 105 °C for 2 h (which has not been proved to cause thermochemical degradation of NSCs) to denature the enzymes and at 65 °C to constant weight for 48 h and weighed.

### 2.3. Non-Structural Carbohydrates Concentrations

The dried plant samples were ground using a ball mill for the NSC analysis. In this study, the soluble sugars were defined as the sum of fructose, glucose, and sucrose, while the total NSC referred to the sum of the soluble sugar and starch.

The soluble sugars were extracted by adding 80% ethanol solution to 0.5 g of the ground samples and ultrasonically shaken for 30 mins under 65 °C. The extraction was performed twice by adding 70 mL 80% ethanol solution in total to ensure the samples were extracted sufficiently, and the extractant was processed using a rotary evaporator [32,33,34]. The supernatant was diluted to 10 mL with deionized water and filtered using 0.22 µm nylon membranes (Keyilong, Tianjin, China). The filtered extractant was injected into an ion chromatograph that was equipped with an integrated pulse amperometric detection (ICS-5000+, ThermoFisher, San Jose, CA, USA) to separate the three components of soluble sugars. An Ag/AgCl electrode was used as the reference electrode and a gold electrode as the working electrode in the amperometric detection. The CarboPac PA 1 chromatographic column (4 × 250 mm) was used as the separation column, in which a 2 mol/L NaOH solution was used as the mobile phase with a flow rate of 1 mL/min. During the detection, the separation of each sample lasted 10 min, and the temperature of the column was maintained at 30 °C.

The starch was extracted according to the perchloric acid extraction method [33]. Briefly, the starch was extracted by first adding 10 mL of 9.2 M HClO_4_ and subsequently 10 mL of 4.6 M HClO_4_ to the gelatinized residue (left after extracting the soluble sugars). The extractant was then diluted to 50 mL for the long time preservation. The starch concentration was detected by the anthrone colorimetric method using a spectrophotometer (SMB80-3003-76, Sartorius stedim biotech, Goettingen, Germany) at 620 nm. The solution was additionally diluted 10 times before the detection. 

### 2.4. Statistical Analyses

Two-way repeated measures analysis of variance (ANOVA) were used to compare the effects of the duration (time) and intensities of the drought treatments on photosynthesis, biomass, the concentrations of glucose, fructose, sucrose, soluble sugar, starch and total NSC, and the ratio of soluble sugar to starch. The first time sampling is the control for the duration, which was established on the day before treatment started (initial) and represents the initial status of the seedlings. As for drought intensity, we sampled 1 seedling that grew under field capacity (control) from each block (4 in total) on each sampling date. Mauchly’s sphericity test was applied to examine the assumption of sphericity [35]. If there was no significant interaction, only the primary effects of time and/or drought intensities were examined separately. The least significant difference (LSD) method was used for the multiple comparisons (*p* < 0.05). The statistical analyses were performed using SPSS v.24.0 (IBM Inc., Armonk, NY, USA). *F*-statistic and probabilities (*p*) from repeated-measure analysis of variance were shown in Table S2.

## 3. Results

### 3.1. Photosynthetic Characteristics and Biomass under Different Drought Treatments

**Figure 1:**
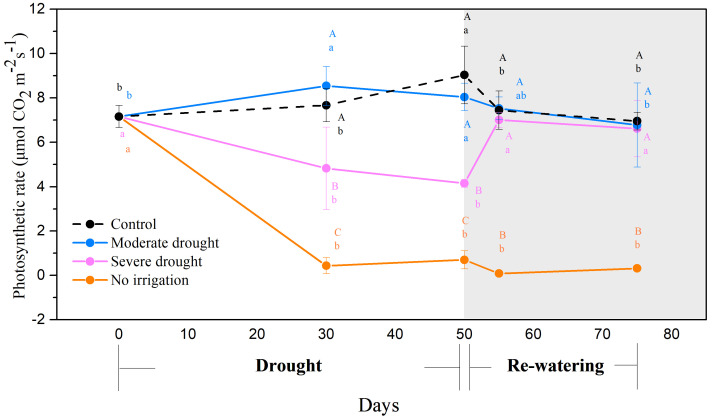
Photosynthetic rate during drought (0–50 days) and the subsequent re-watering (50–75 days) for *P. tabulaeformis* seedlings in the control (black line), moderate drought (blue line), severe drought (purple line), and no irrigation (orange line) groups. Values are means ± SD (*n* = 4). The different uppercase letters represent the significant differences among the four treatments (including control) on the same sampling day. The different lowercase letters represent the significant differences between the sampling days under the same treatment.

After 30 days, no irrigation and severe drought treatments significantly decreased photosynthetic rates (Figure 1), stomatal conductance (Figure S1a), and transpiration rates (Figure S1b) by more than 90% under the no-irrigation condition and more than 30% under severe drought condition compared with the control (*p* < 0.05). However, no significant difference was found between the moderate drought group and the control. The three photosynthetic characteristics (i.e., photosynthetic rates, stomatal conductance, and transpiration rates) did not change significantly under the no-irrigation treatment, even after re-watering. In contrast, all three photosynthetic characteristics were recovered to the control level within five days for the severe drought treatment. Overall, both the no irrigation and severe drought treatments significantly reduced the photosynthesis of the seedlings, but the photosynthetic rates only recovered after re-watering under the severe drought treatment.

The biomass of the leaves, twigs, and roots in the control significantly increased over the course of the experiment, especially for the leaves and roots (Table 1, *p* < 0.05). However, after 50 days, the no-irrigation treatment significantly inhibited the growth of *P. tabulaeformis* seedlings, especially the leaves, whose biomass remained nearly unchanged and was 30.9% lower than the control (Table 1, *p* < 0.05). From 50 to 75 days, the biomass of the leaves and roots under no irrigation and severe drought treatments did not show any sign of significant increase during re-watering. After 75 days, the leaf and root biomass under no irrigation and severe drought treatments were 52.0%, 70.2% and 58.3%, 66.3% of the controls, respectively (*p* < 0.05).

### 3.2. The NSCs Concentrations in Different Organs during Drought and Re-Watering

#### 3.2.1. The NSCs Concentrations in the Leaves

**Figure 2:**
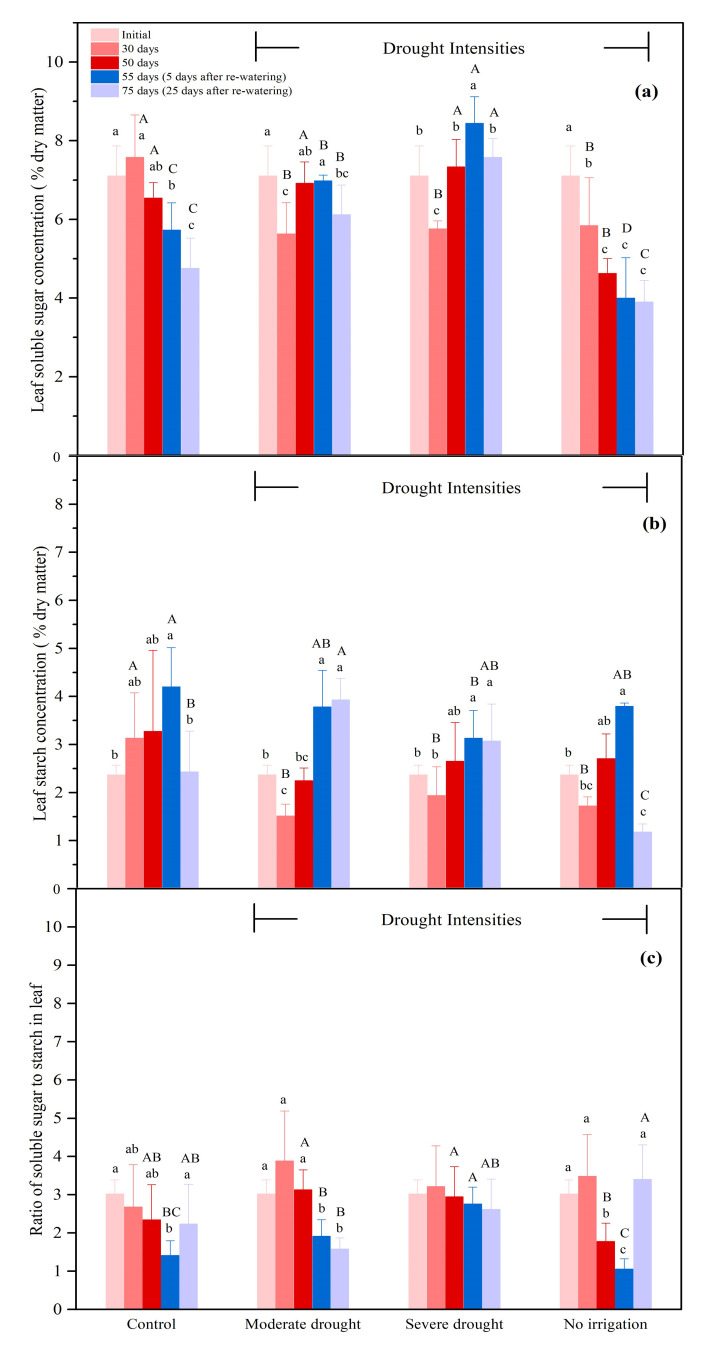
Soluble sugar concentration (**a**), starch concentration (**b**), the ratio of soluble sugar to starch (**c**) in the leaves during drought (0–50 days), and the subsequent re-watering (50–75 days) for *P. tabulaeformis* seedlings in the control, moderate drought, severe drought, and no irrigation groups. In this study, soluble sugar is considered as the sum of glucose, fructose, and sucrose. Values are the means ± SD (*n* = 4). The different uppercase letters represent the significant difference among the four treatments (including control) on the same sampling day. The different lowercase letters represent the significant differences between the sampling days under the same treatment.

There were some decreases in soluble sugar concentration initially in moderate and severe drought, but only the no-irrigation treatment showed consistent decreases in soluble sugar over time. Re-watering did not show any consistent effect on soluble sugar. (Figure 2a). After 30 days, the concentration of the soluble sugar in the leaves under no irrigation, severe, and moderate drought conditions were 77.1%, 76.0%, and 74.3% of the control, respectively (Figure 2a, *p* < 0.05). The sucrose concentration of the leaves under the no-irrigation condition was significantly higher than that under severe and moderate drought conditions (Figure S2a, *p* < 0.05). From 55 to 75 days, the leaf soluble sugar concentration in the control also decreased by 17.0% (*p* < 0.05). As for the severe and moderate drought treatments, similar to the control, re-watering significantly decreased the concentration of leaf soluble sugar concentration from 55 to 75 days by 10.3% and 12.3%, respectively (Figure 2a, *p* < 0.05), while no significant change was detected under the no-irrigation treatment (Figure 2a). There was no consistent pattern of starch concentration under different drought treatments. After re-watering, only the control and no-irrigation treatment showed a significant drop in starch concentrations (can see the increase in ratio in Figure 2c). After 30 days, the leaf starch concentration under no irrigation, severe, and moderate drought conditions were 54.9%, 61.9%, and 48.3% of the control, respectively (Figure 2b, *p* < 0.05). From 55 to 75 days, the leaf starch concentration decreased by 42.2% in the control (Figure 2b, *p* < 0.05). It is different from the control that there was no significant change detected for the leaf starch concentration under severe and moderate drought treatment after re-watering (Figure 2b). The significantly decreased starch concentration under the no-irrigation treatment resulted in a significant increase in the ratio of soluble sugar to starch from 55 to 75 days (Figure 2b,c, *p* < 0.05). In terms of the total NSC concentration, seedlings under no irrigation, severe, and moderate drought conditions all exhibited a significant reduction in total NSC concentration up to 30 days (Figure S3a, *p* < 0.05). After 75 days, the total NSC concentration under the no-irrigation treatment was significantly lower than the other groups (severe, moderate drought, and control) due to the reduced leaf starch concentration (Figure S3a, *p* < 0.05). Additionally, the total NSC concentration in control was decreased by 27.6% from 55 to 75 days (Figure S3a, *p* < 0.05). 

#### 3.2.2. The NSCs Concentrations in the Twigs

**Figure 3:**
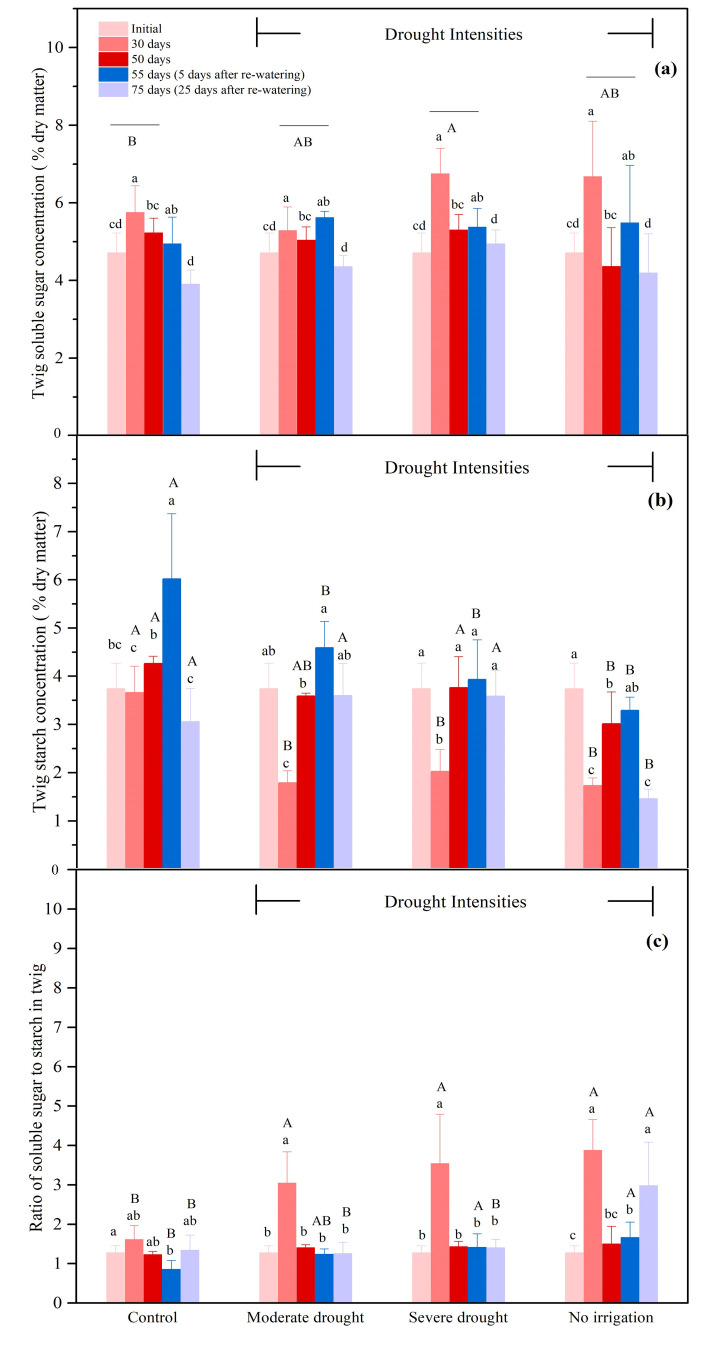
Soluble sugar concentration (**a**), starch concentration (**b**), the ratio of soluble sugar to starch (**c**) in the twigs during drought (0–50 days), and the subsequent re-watering (50–75 days) for *P. tabulaeformis* seedlings in the control, moderate drought, severe drought, and no irrigation groups. In this study, soluble sugar is considered as the sum of glucose, fructose, and sucrose. Values are the means ± SD (*n* = 4). The different uppercase letters represent the significant difference among the four treatments (including control) on the same sampling day. The different lowercase letters represent the significant differences between the sampling days under the same treatment.

Within twigs, drought resulted in an increase in soluble sugar concentration while starch concentration tended to decrease. Specifically, after 30 days, the concentration of the soluble sugar in the twigs increased under all treatments, with a larger magnitude of increment under no irrigation and severe drought conditions in comparison with that of the control (Figure 3a). After 30 days, the starch concentration under the no irrigation, severe, and moderate drought conditions were 47.2%, 55.3%, and 48.8% of the control, respectively (Figure 3b, *p* < 0.05). From 55 to 75 days, surprisingly, starch concentration did not recover after re-watering in either the control or the no irrigation treatment. In the meantime, there was no significant reductions in starch concentration under severe and moderate drought treatments after re-watering, which was different from the control (Figure 3b). Up to 30 days, the opposite responses of soluble sugar and starch concentrations further contributed to the significant increase of the ratio of soluble sugar to starch, which were 240.6%, 219.9%, and 188.9% of the control under no irrigation, severe, and moderate drought conditions, respectively (Figure 3c, *p* < 0.05). 

#### 3.2.3. The NSCs Concentrations in the Stems

**Figure 4:**
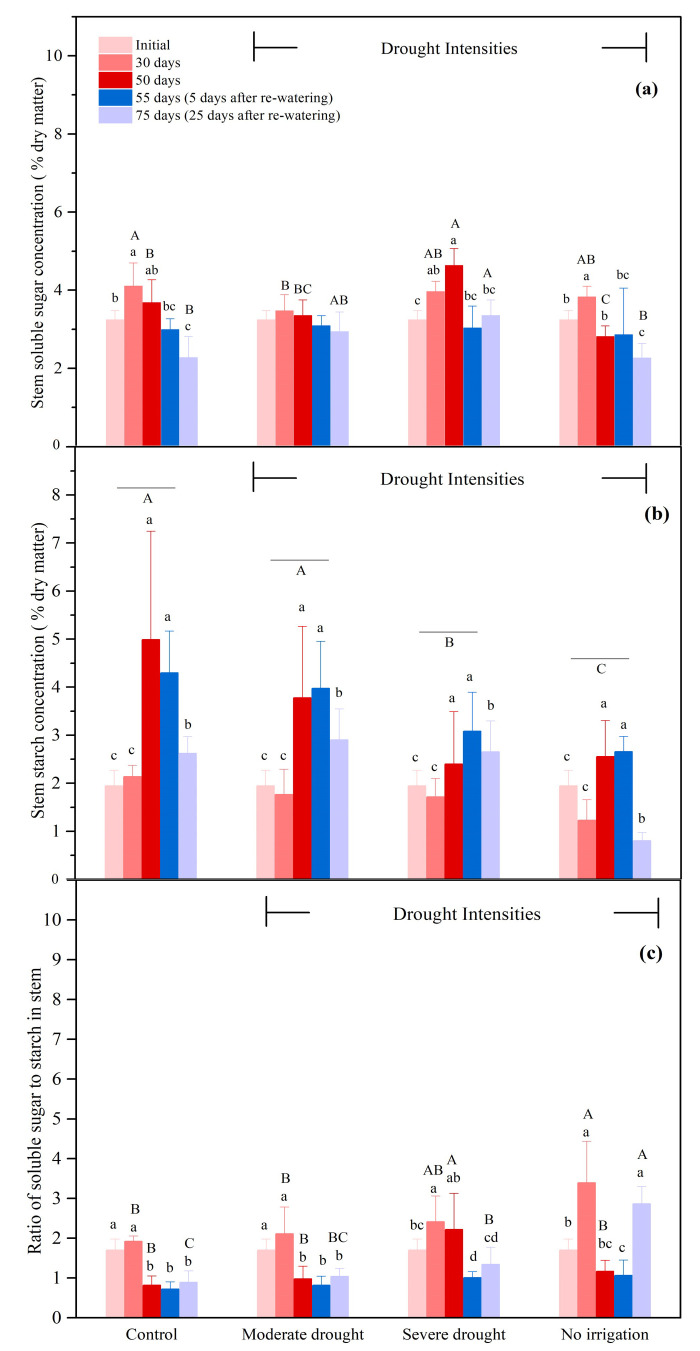
Soluble sugar concentration (**a**), starch concentration (**b**), the ratio of soluble sugar to starch (**c**) in the stems during drought (0–50 days), and the subsequent re-watering (50–75 days) for *P. tabulaeformis* seedlings in the control, moderate drought, severe drought, and no irrigation groups. In this study, soluble sugar is considered as the sum of glucose, fructose, and sucrose. Values are the means ± SD (*n* = 4). The different uppercase letters represent the significant difference among the four treatments (including control) on the same sampling day. The different lowercase letters represent the significant differences between the sampling days under the same treatment.

Soluble sugar concentrations in stem were also affected in the control (in addition to severe and no irrigation, Figure 4a). During drought, the starch concentration in the stems was only affected by the severe and no irrigation treatments (Figure 4b). The starch concentration in the stems under no irrigation and severe drought conditions was 57.5% and 80.4% of the control, respectively, after 30 days, and 51.2% and 48.0% of control, respectively, after 50 days (Figure 4b, *p* < 0.05). From 55 to 75 days, the starch concentration in the stems was reduced by 39.1% in the control (Figure 4b, *p* < 0.05). Similar to the control, the starch concentration in the stem was reduced by 69.8% under the no-irrigation treatments after re-watering and it was significantly lower than that in the other groups (Figure 4b, *p* < 0.05). The increased concentration of soluble sugar and reduced starch concentration contributed to the increased ratio of soluble sugar to starch under the no-irrigation condition after 30 days, which was 176.6% of the control (Figure 4c, *p* < 0.05). None treatment showed a significant increase in total NSC concentration at 30 days. 

#### 3.2.4. The NSCs Concentrations in the Roots

**Figure 5:**
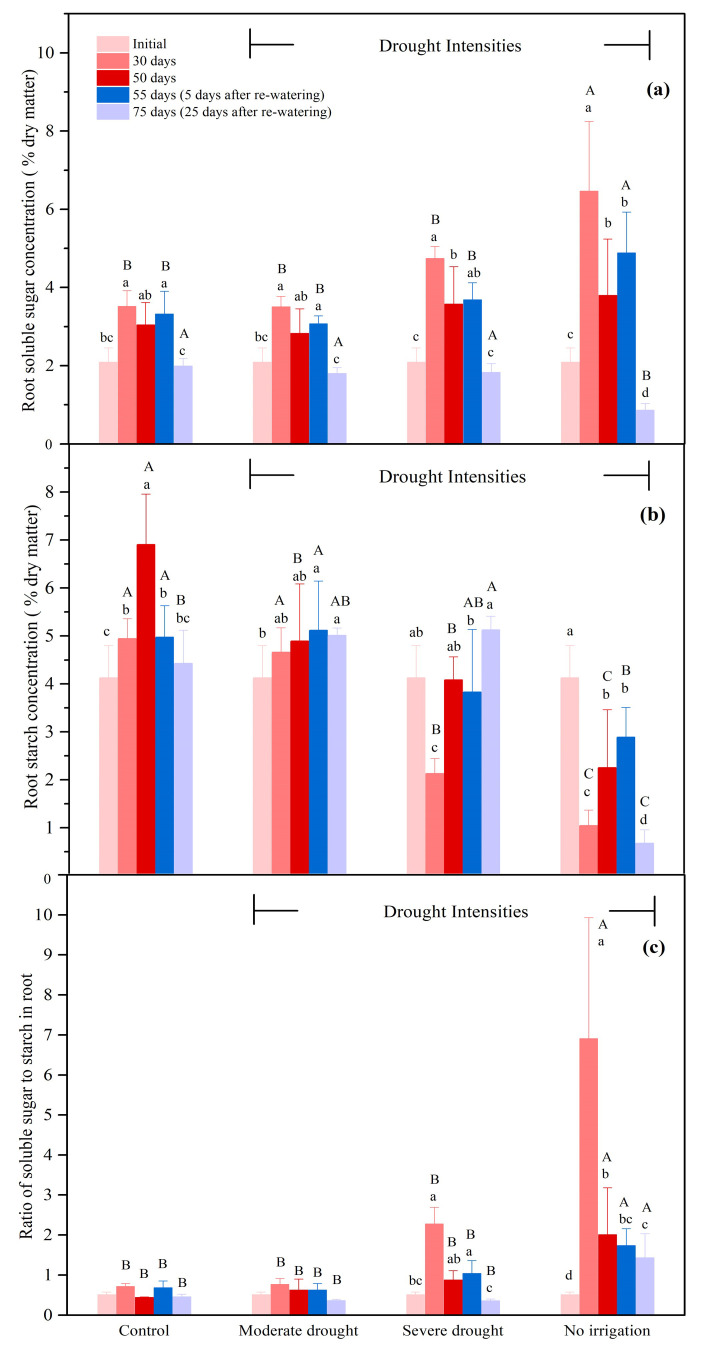
Soluble sugar concentration (**a**), starch concentration (**b**), the ratio of soluble sugar to starch (**c**) in the roots during drought (0–50 days), and the subsequent re-watering (50–75 days) for *P. tabulaeformis* seedlings in the control, moderate drought, severe drought, and no irrigation groups. In this study, soluble sugar is considered as the sum of glucose, fructose, and sucrose. Values are the means ± SD (*n* = 4). The different uppercase letters represent the significant difference among the four treatments (including control) on the same sampling day. The different lowercase letters represent the significant differences between the sampling days under the same treatment.

Drought stress induced a strong increase in the ratio of soluble sugar to starch in roots, caused by the strong increase in soluble sugar concentration and decline in starch concentration. After re-watering, the seedlings in the no-irrigation treatment showed a simultaneous decrease in soluble sugar and starch concentration, while the severe and moderate drought treatments only showed decreases in soluble sugars (not starch). After 30 days, the soluble sugar concentration in the roots under the no-irrigation condition was 183.9% of the control (Figure 5a, *p* < 0.05). In addition, the soluble sugar concentration in the roots under the severe drought condition was also slightly higher (though not significant) than that of the control at 30 days (Figure 5a). The change of soluble sugar concentration in roots was larger under the no-irrigation condition than under the severe drought condition. After re-watering, the soluble sugar concentration in the seedlings that experienced no irrigation was reduced by 82.4% from 55 to 75 days and it was significantly smaller than that under severe and moderate drought treatments (Figure 5a, *p* < 0.05). Meanwhile, a 40.0% decrease of the root soluble sugar concentration was observed for the control. The starch concentration in the roots was significantly depressed by drought stress. The starch concentration in the roots under no irrigation and severe drought conditions were 21.1% and 43.0% of the control, respectively, after 30 days, and 32.6% and 59.1% of the control, respectively, after 50 days (Figure 5b, *p* < 0.05). Similar to soluble sugars, the change of starch concentration in roots was also larger under the no-irrigation condition than under the severe drought condition. From 55 to 75 days, a 76.6% reduction of starch concentration in the roots was found under the no-irrigation treatment (Figure 5b, *P* < 0.05). Conversely, from 55 to 75 days, the starch concentration in the roots increased by 34.0% under the severe drought treatment (*p* < 0.05) and stayed stable under the moderate drought treatment after re-watering (Figure 5b). As for the ratios of soluble sugar to starch in the roots, significant increases were observed under no irrigation and severe drought conditions up to 30 days, because of the reduced starch concentration and increased soluble sugar concentration (Figure 5c, *p* < 0.05). In contrast, the ratio of soluble sugar to starch in the control remained stable over the course of the entire experiment (Figure 5c). In addition, from 55 to 75 days, the total NSC concentration in the control decreased by 22.6% (Figure S3d, *p* < 0.05). Similar to the control, in the meantime, the simultaneous reduction in the concentrations of soluble sugar and starch led to an 80.2% reduction in the total NSC concentration under the no-irrigation treatment after re-watering (Figure S3d, *p* < 0.05). 

## 4. Discussion

### 4.1. NSC Dynamics under Drought Conditions

These findings generally supported the first hypothesis that drought stress would decrease the starch concentration while increase the concentration of soluble sugar. No irrigation and severe drought treatments largely constrained the photosynthetic C assimilation. The restricted C supply cannot satisfy the C demand of seedlings for maintaining metabolism and growth. The continuous respiration demand of roots likely made itself a strong C sink during drought [36], and plants may have allocated more NSCs from source organs (i.e., leaves) to roots [37]. This was shown by the increased total NSC concentration in roots and decreased total NSC concentration in leaves after 30 days of drought. Besides, the xylem vessels in roots are more prone to be damaged by embolism, so more soluble sugars are required for osmotic adjustment in roots, such as for the refilling of xylem ducts [38,39]. By increasing the allocation of NSCs to the roots, seedlings are likely to maximize the ability of water uptake for survival under drought conditions [40]. In terms of the dynamics of soluble sugars and starch, diverse responses to drought stress were found among the different organs. As drought progressed, the simultaneously decreased soluble sugar and starch concentrations in leaves resulted in a significant decrease in the total NSC concentration after 30 days. This was not only due to the C consumption in leaves, but also the drought-promoted re-allocation of NSCs from the leaves to other organs, especially to the roots [37]. In contrast, the soluble sugar concentration increased significantly in the twigs, roots, and stems, where starch concentrations declined. This feature might be attributed to the conversion of stored starch to soluble sugars [2]. During drought, more soluble sugars are required for metabolism because drought inhibits photosynthesis and reduces C assimilation but does not significantly reduce respiration [3,41]. Sufficient soluble sugars are also required for osmotic regulation to maintain the phloem turgor and maintain phloem transportation and refilling of the xylem embolism under drought stress [21,42]. However, the limited water supply directly induced the stomatal closure and restricted photosynthetic C gain, causing plants to largely rely on consuming their starch storage to satisfy metabolism [9,22,42]. This was demonstrated by the increased ratio of soluble sugar to starch in the twigs, roots, and stems in this study.

The effects of the different drought intensities on the NSCs dynamics were primarily observed in the leaves and roots. The sucrose concentration in the leaves under the no-irrigation condition was significantly higher than that under the severe and moderate drought conditions after 30 days of drought treatment. These findings are consistent with Hartmann et al. (2013), who reported that Norway spruce seedlings accumulated sucrose in the needles and branches under a drought environment. Although glucose and fructose are more efficient agents for osmotic regulation [10], the conversion from sucrose to glucose and fructose was inhibited due to water shortage under severe drought conditions, because water is required for amylase and invertase to catalyze the hydrolytic reactions [36,43]. Besides, Hartmann et al. (2013) have attributed the sucrose accumulation to the hydraulic failure that impeded the long-distance transport of sucrose and finally led to the sucrose accumulation in leaves. Moreover, the accumulated sucrose in the leaves can also provide energy to maintain cell survival [44]. The changes of soluble sugar and starch concentrations were stronger under the no-irrigation condition than under the severe drought condition in roots but not in the stems and twigs. This is additional evidence indicating that the roots are more sensitive to drought, as we mentioned above. As the drought stress was intensified, the roots tended to convert more starch storage to satisfy the greater needs of soluble sugars.

### 4.2. NSCs Dynamics during Re-Watering

Different drought intensities had different effects on the seedlings after the drought stress was reduced. The failure of photosynthesis recovery of seedlings under the no-irrigation condition resulted in the serious consumption of NSC, especially in roots, which is almost completely depleted. The seedlings that experienced severe and moderate droughts before re-watering had strong tendencies to maintain and rebuild their starch storage in all the organs, especially the leaves and roots, during re-watering, which was significantly different from the seedlings that did not suffer drought stress (i.e., the control).

Our findings were partially inconsistent with the second hypothesis that the starch concentration would significantly decrease in the roots after re-watering. The starch, as well as the total NSC concentration in the leaves and roots under the severe and moderate drought conditions, did not show any decreasing tendency during re-watering, which was contrary to the seedlings that never experienced the drought treatments (i.e., the control), which showed a significant reduction in the starch and total NSC concentrations in all the organs from 50 to 75 days (*p* < 0.05). As the biomass of leaves and roots were significantly increased in the control from 50 to 75 days, we speculate that the reduced NSC was actively allocated to support the respiration and growth of seedlings in the control. On the contrary, even though the photosynthesis of seedlings under severe drought conditions quickly recovered within five days after re-watering, the biomass of the leaves and roots did not increase significantly over the course of re-watering. This delayed growth recovery was also observed in previous studies, indicating that plants cannot preferentially recover growth after re-watering [45,46,47,48]. This phenomenon is probably associated with the priority of storage over growth. Drought can impart delayed effects for plants after drought relief, which is called the “drought legacy effect”, and this might last for several years [1]. By using the isotope labeling technique, Galiano et al. (2017) showed that previously drought-stressed seedlings of *P. sylvestris* preferentially invested most of the newly assimilated C to storage and osmotic protection after re-watering, while the newly assimilated C was mostly invested in growth in the well-watered seedlings [49]. Therefore, it was speculated that the rapidly recovered C assimilation after re-watering in seedlings under the severe drought condition might be mostly invested in starch storage rather than growth, as indicated by the accumulated leaf and root starch concentrations and the nearly constant biomass of the leaves and roots of seedlings during 25 days of re-watering [50]. This phenomenon of priority for storage and delayed growth, which did not appear in the seedlings from the control, confirmed the “drought legacy effect” that was mentioned above. In this study, the moderate drought treatment also affected the NSCs dynamics during the drought and re-watering periods, whereas the it did not significantly affect photosynthesis and the growth of seedlings. Compared to seedlings that have never experienced drought stress (i.e., the control), the mechanism of storing NSCs during the re-watering of seedlings that have experienced moderate drought before may be an advantage for survival in the future under further environmental stresses.

After re-watering, the photosynthesis and the dynamics of the NSCs in all the organs of the seedlings that experienced the no-irrigation treatment showed a different responses compared to those under severe and moderate drought conditions. Similar to the variation in NSC concentration in the control, the seedlings under the no-irrigation treatment consumed their starch storage during re-watering, but without recovering photosynthesis, which partially supports the second hypothesis. Anderegg et al. (2012) proposed the “point of no return” concept, which refers to an irreversible state where plant organs are dying, although biological activities can still be detected after releasing the drought stress [51,52]. The recovery of such fatal damage that induced the irreversible state of seedlings can only be accomplished by regrowth of new tissues, with the precondition of sufficient C supply and the functioning apical and cambial meristematic tissues [25]. In this study, the photosynthesis of seedlings did not recover, as the heavy consumption of starch storage under the no-irrigation treatment could not satisfy the C demand for regrowth of the new tissues, resulting in the irreversible state of seedlings, even if the drought stress was relieved. Previous studies have also demonstrated that irreversible hydraulic failure induced by drought strongly jeopardized phloem transportation, resulting in a transport failure between the aboveground organs and roots [36,53,54]. Therefore, the roots can only rely on the internal NSCs to maintain metabolism until the root C reserves are eventually exhausted, which probably results in C starvation in roots under severe drought circumstances [36,53,54]. The findings of this study provide strong evidence for the C starvation of roots, as both the soluble sugar and starch concentration under the no-irrigation condition were significantly reduced in roots after re-watering.

However, in this study, only the no-irrigation treatment induced fatal damage to the seedlings. In contrast, there was no irreversible damage on seedlings observed under severe and moderate drought conditions, indicating the strong drought resistance of *P. tabulaeformis*.

### 4.3. Limitations of the Experimental Methods

Although our current study provided strong evidence that NSCs, especially starch storage, play a key role in the resistance and resilience of *P. tabulaeformis* seedlings under drought stress, with adequate experimental setup, there were still some limitations in this study. First, the re-watering process was only conducted for 25 days, and this may overlook any possible mid- to long-term responses. The maintenance of starch storage under the severe and moderate drought conditions may only reflect a short-term effect after re-watering, and seedlings may begin to consume starch storage by regrowth over a longer time period. Second, this experiment was conducted using artificially imposed drought and re-watering in the pots in a greenhouse, where field conditions, including variable environment conditions (variable temperature, irradiation intensity, and CO_2_ concentrations), allelopathy, and interactions between single seedlings, could not be fully simulated [25]. In addition, the natural re-watering (rainfed) process was gradually achieved, but we conducted the re-watering by immediate thorough irrigation in a pot in a short time, which may have caused a sudden reaction in the seedlings. Third, this experiment was conducted on immature three-year-old seedlings of *P. tabulaeformis.* Mature trees have greater C reserves with longer transport distances for water and nutrients compared to seedlings. It is likely that mature trees in the field would provide more comprehensive understanding of drought-induced NSC allocation under a long-time scale. But the survival of seedlings is as important as that of mature trees, as seedling survival is a key step in forest regeneration. Fourth, we can only infer the pattern of NSC allocation from leaves to roots based on the observed variation in total NSC concentration and extant scientific consensus from literature. Further studies on the allocation mechanism of NSCs are merited by the isotope labeling technique. Moreover, due to the heavy workload, we only implemented four replicates and more replicates would be useful to improve the statistical robustness. Finally, in these experiments, although it was found that NSC storage was restored, it was not shown whether such processes were active or passive. The latter is mostly due to the sink limitation, which is primarily caused by a deficient nutrient supply (e.g., nitrogen limitation), temperature, and water inhibition on the rate of biosynthesis and development [55], while the former storage process is regulated by genes [8]. Starch storage is an important C sink in plants that plays an important role when plants are suffering environmental stress. How NSC storage is built up has become a vigorous debate in recent years. Therefore, further research is required to investigate the nutritional and hydraulic status of plants under drought stress, along with some genetic and enzyme investigations of the metabolism process that involves active and passive storage [8,56].

## 5. Conclusions

This study provided unique observations and valuable information on how and why the non-structural carbohydrate concentrations in each organ of *Pinus tabulaeformis* seedlings changed under different intensities of drought and re-watering. It was found that starch storage played an important role in the seedling resistance to drought stress and recovery after re-watering. During drought, more NSCs were allocated to roots of *P. tabulaeformis* seedlings and the starch storage was depleted in each organ (leaves, twigs, stems, and roots) to fuel the needs of respiration, osmotic regulation, and hydraulic repair of the plants due to the reduction of C assimilation and the lack of water. During re-watering, the seedlings did not consume the starch storage to promote hydraulic repair and regrowth under the severe and moderate drought treatments, as was hypothesized. However, starch storage remained stable or even increased at the expense of growth in the leaves and roots due to the “drought legacy effect”. It was also found that only the no-irrigation treatment caused irreversible damage to the seedlings, but not severe and moderate drought, suggesting that *P. tabulaeformis* is a suitable species for afforestation and soil and water conservation in arid areas owing to its strong drought resistance and resilience. Further investigation of the NSC dynamics for different tree species under various environmental stresses would certainly advance our understanding of the underlying mechanisms of NSC allocation strategies and provide new insights for the selection of forest tree species for different geographic regions.

## Figures and Tables

**Table 1:** Biomass (g) of the leaves, twigs, stems, and roots under the four treatments (including control) of *P. tabulaeformis* seedlings during drought (0–50 days) and subsequent re-watering (50–75 days). The change in the biomass was expressed as dry weight, and values are the means ± SD (*n* = 4).

Organ	Treatment	0 Day (Initial)	50 Days	75 Days (25 Days after Re-Watering)
**Leaf**	Control	13.898(3.538) ^A c^	17.653(1.867) ^AB b^	23.620(3.108) ^A a^
	Moderate drought	13.898(3.538) ^A b^	12.994(1.459) ^BC b^	24.151(4.801) ^A a^
	Severe drought	13.898(3.538) ^A b^	20.978(5.717) ^A a^	16.579(1.837) ^B ab^
	No irrigation	13.898(3.538) ^A a^	12.194(2.672) ^C a^	12.271(2.022) ^B a^
**Twig**	Control	1.593(1.009) ^A b^	2.457(1.070) ^A a^	3.923(0.568) ^A a^
	Moderate drought	1.593(1.009) ^A b^	2.833(0.676) ^A a^	3.013(0.693) ^A a^
	Severe drought	1.593(1.009) ^AB b^	3.037(0.983) ^AB a^	2.660(0.617) ^AB a^
	No irrigation	1.593(1.009) ^A b^	1.583(0.406) ^A a^	1.950(0.310) ^A a^
**Stem**	Control	15.777(6.436) ^A a^	19.243(3.113) ^A a^	19.523(0.846) ^A a^
	Moderate drought	15.777(6.436) ^A a^	17.837(6.898) ^A a^	16.780(2.973) ^A a^
	Severe drought	15.777(6.436) ^A a^	17.260(0.877) ^A a^	15.477(5.413) ^A a^
	No irrigation	15.777(6.436) ^A a^	16.467(3.974) ^A a^	16.167(1.685) ^A a^
**Root**	Control	9.349(1.239) ^A b^	9.342(0.729) ^A b^	12.926(1.099) ^A a^
	Moderate drought	9.349(1.239) ^A b^	11.204(5.173) ^A ab^	12.250(1.319) ^A a^
	Severe drought	9.349(1.239) ^A a^	8.977(1.889) ^A a^	8.566(0.887) ^B a^
	No irrigation	9.349(1.239) ^A a^	7.020(1.051) ^A b^	7.538(1.486) ^B b^

The different uppercase letters represent the significant differences among the four treatments (including control) on the same sampling day. The different lowercase letters represent the significant differences between the sampling days under the same treatment.

## Data Availability

Data are available on request due to restrictions, e.g., privacy or ethical. The data presented in this study are available on request from the corresponding author. The data are not publicly available due to the strict management of various data and technical resources within the research teams.

## Supplementary Materials

The following are available online at https://www.mdpi.com/article/10.3390/biology10040281/s1, Figure S1: Stomatal conductance (a), and transpiration rate (b) during drought (0-50 days) and the subsequent re-watering (50–75 days) for P. tabulaeformis seedlings in the control (black line), moderate drought (blue line), severe drought (purple line), and no irrigation (orange line) groups, Figure S2: Soluble sugars concentration in the leaves(a), twigs(b), stems(c) and roots(d) during drought (0–50 days) and the subsequent re-watering (50–75 days) for P. tabulaeformis seedlings in the control, moderate drought, severe drought, and no irrigation groups, Figure S3: Total NSC concentration in the leaves(a), twigs(b), stems(c) and roots(d) during drought (0–50 days) and the subsequent re-watering (50–75 days) for P. tabulaeformis seedlings in the control, moderate drought, severe drought, and no irrigation groups, Table S1: Soil water content (%) in the four treatments (including control) of P. tabulaeformis seedlings during drought (0–50 days) and subsequent re-watering (50–75 days), Table S2: F-statistic and probabilities (p) from repeated-measure analysis of variance on glucose, fructose, sucrose, soluble sugar, starch, total non-structural carbohydrate (NSC) and the ratio of soluble sugar to starch (soluble sugar: starch) in different seedling tissues.
