# Supplementary material for: The Effects of Drought and Re-Watering on Non-Structural Carbohydrates of Pinus tabulaeformis Seedlings
Source: Biology (Basel). 2021 Mar 30;10(4):281. doi: 10.3390/biology10040281 (PMC8066268; doi:10.3390/biology10040281)
Supplement: Supplementary file 1 [file biology-10-00281-s001.pdf]

biology

MDPI

Supplementary Materials

The Effects of Drought and Re-watering on Non-structural Carbohydrates of *Pinus tabulaeformis* Seedlings

Xinyi Guo, Changhui Peng, Tong Li, Jingjing Huang, Hanxiong Song, Qiuan Zhu and Meng Wang

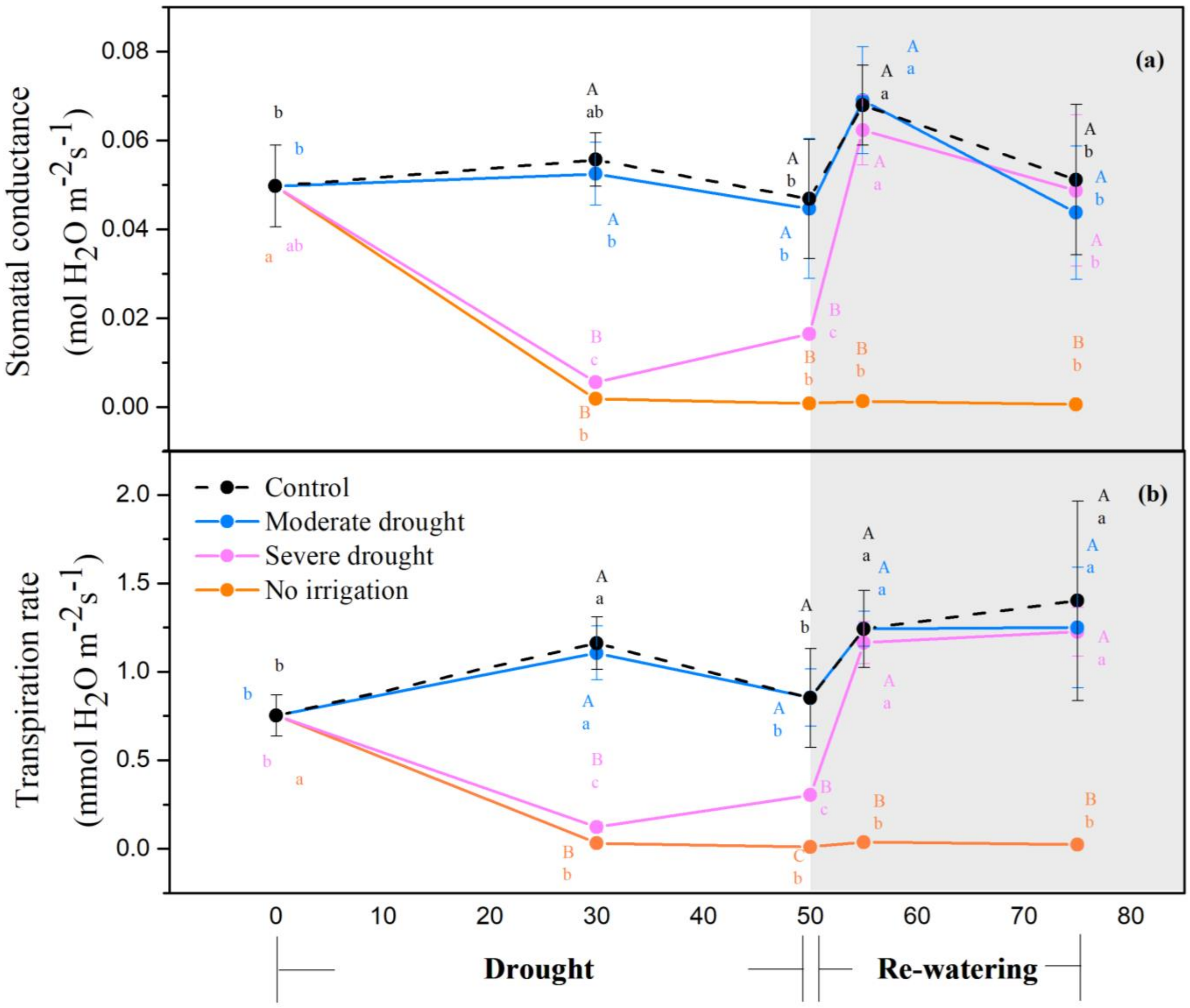

Figure S1. Stomatal conductance (**a**), and transpiration rate (**b**) during drought (0-50 days) and the subsequent re-watering (50-75 days) for *P. tabulaeformis* seedlings in the control (black line), moderate drought (blue line), severe drought (purple line), and no irrigation (orange line) groups. Values are means ± SD (n = 4). The different uppercase letters represent the significant difference among the four treatments (including control) at the same sampling day. The different lowercase letters represent the significant differences between the sampling days under the same treatment.

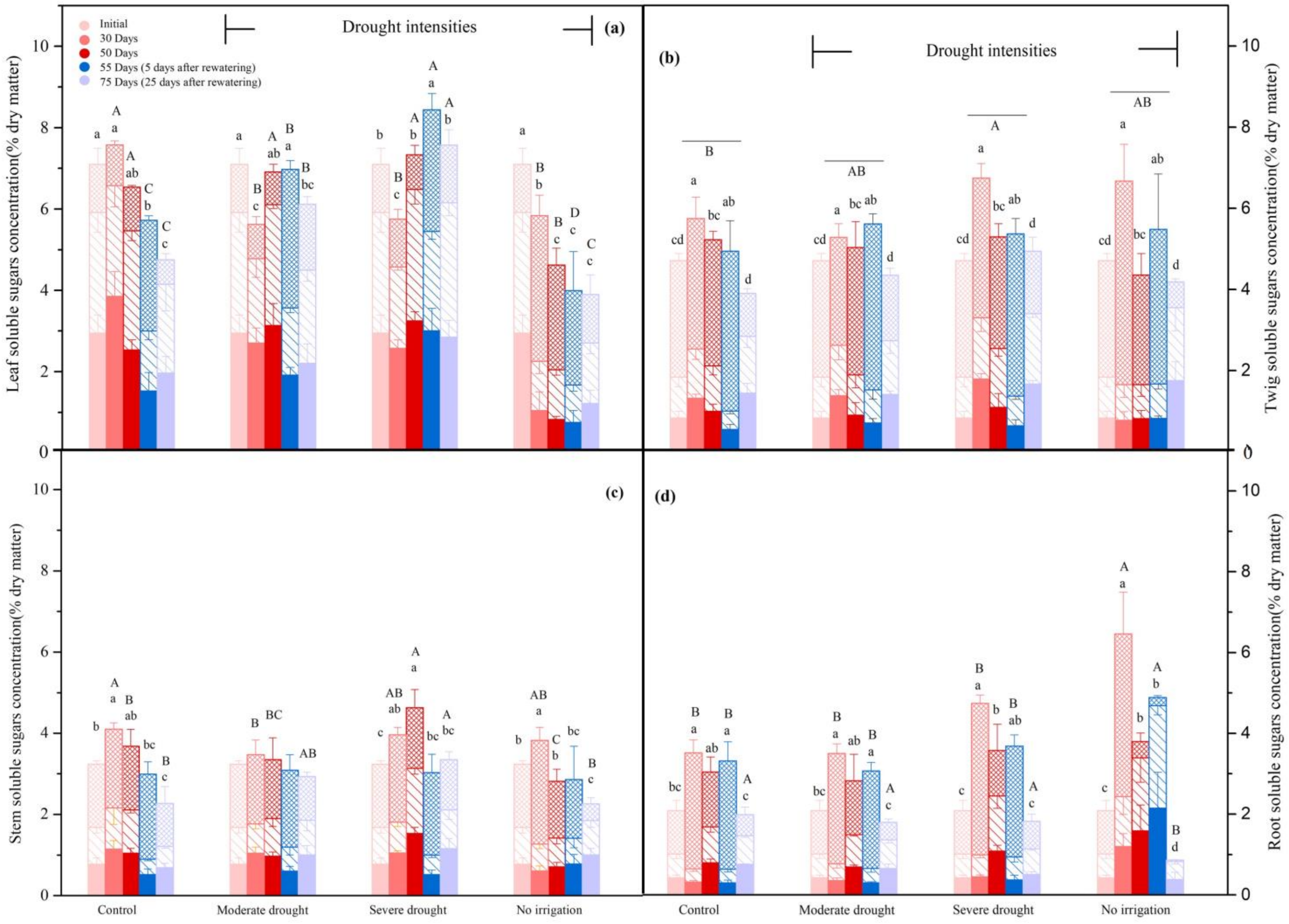

Figure S2. Soluble sugars concentration in the leaves(**a**), twigs(**b**), stems(**c**) and roots(**d**) during drought (0-50 days) and the subsequent rewatering (50-75 days) for *P. tabulaeformis* seedlings in the control, moderate drought, severe drought, and no irrigation groups. The color filled rectangle in each histogram represents the glucose concentration, the slash filled rectangle represents the fructose concentration, and the grid filled rectangle represents the sucrose concentration. In this study, soluble sugar is considered as the sum of glucose, fructose, and sucrose. Values are the means ± SD (n = 4). The different uppercase letters represent the significant difference among the four treatments (including control) on the same sampling day. The different lowercase letters represent the significant differences between the sampling days under the same treatment.

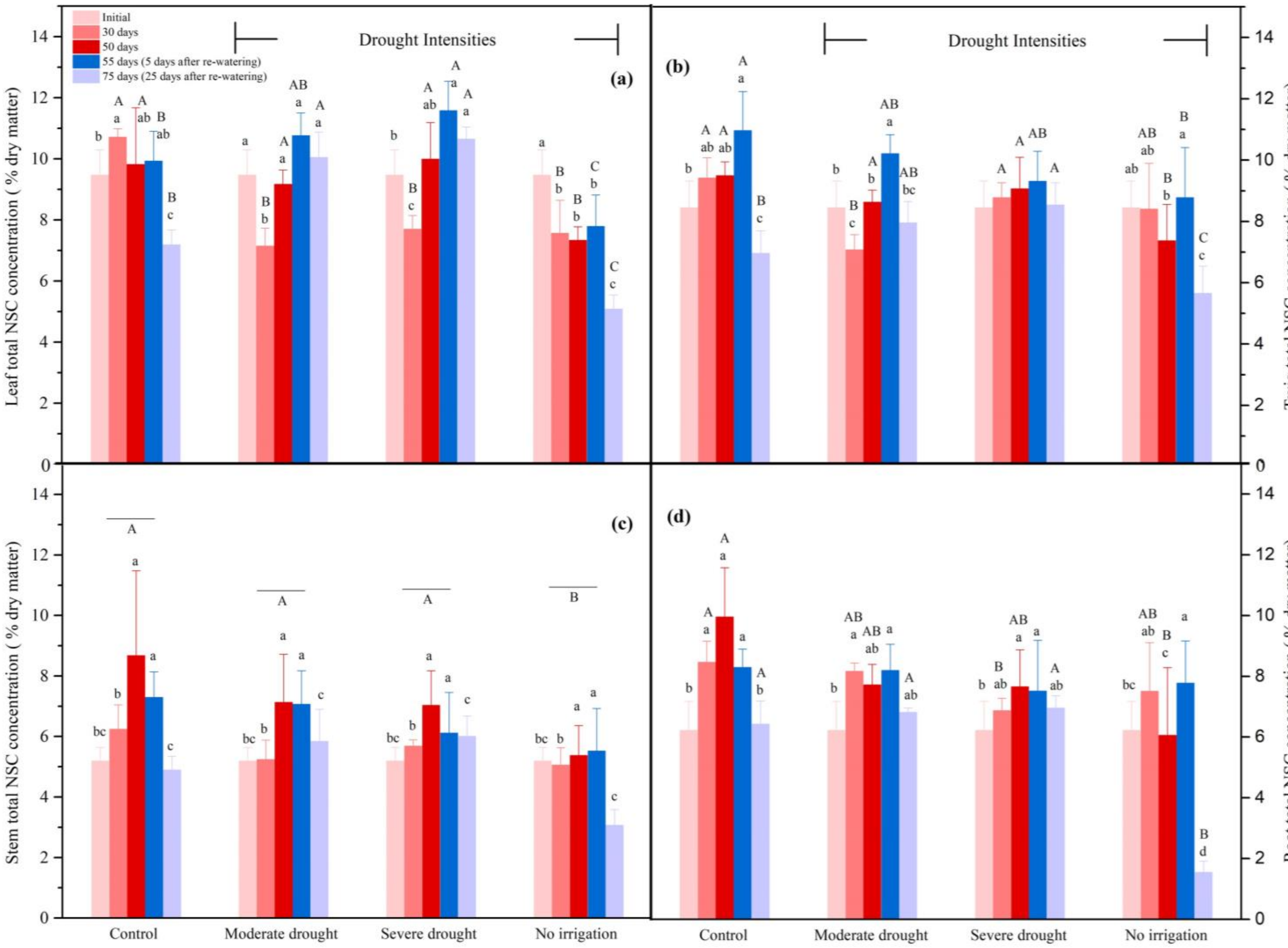

Figure S3. Total NSC concentration in the leaves(**a**), twigs(**b**), stems(**c**) and roots(**d**) during drought (0-50 days) and the subsequent rewatering (50-75 days) for *P. tabulaeformis* seedlings in the control, moderate drought, severe drought, and no irrigation groups. Values are the means ± SD (*n* = 4). The different uppercase letters represent the significant difference among the four treatments (including control) on the same sampling day. The different lowercase letters represent the significant differences between the sampling days under the same treatment.

Table S1. Soil water content (%) in the four treatments (including control) of *P. tabulaeformis* seedlings during drought (0-50 days) and subsequent re-watering (50-75 days). Values are the means ± SD (*n* = 4).

**Treatment**	**0 day (Initial)**	**30 days**	**50 days**	**55 days (5 days after re-watering)**	**75 days (25 days after re-watering)**
Control	25.04(0.91)	26.39(1.05)	25.43(1.41)	25.00(1.64)	25.55(.55)
Moderate drought	25.04(0.91)	13.28(2.42)	10.92(0.96)	25.34(1.87)	26.73(4.15)
Severe drought	25.04(0.91)	4.05(0.59)	4.56(1.04)	25.23(0.35)	26.32(0.13)
No irrigation	25.04(0.91)	1.25(0.13)	1.12(0.03)	26.58(0.08)	28.25(0.92)

Table S2. *F*-statistic and probabilities (*P*) from repeated-measure analysis of variance on glucose, fructose, sucrose, soluble sugar, starch, total non-structural carbohydrate (NSC) and the ratio of soluble sugar to starch (soluble sugar : starch) in different seedling tissues. Columns present the *F* and *P* values of drought intensity, duration and intensity-duration interaction (drought intensity × duration).

**Variables**	**Tissues**	**Drought intensity**		**Duration**		**Drought intensity×Duration**	
		F	***P***	***F***	***P***	***F***	***P***

Glucose	leaves	37.522	< 0.001	22.338	< 0.001	10.968	< 0.001
	twigs	7.034	0.006	46.754	< 0.001	4.106	< 0.001
	stems	21.389	< 0.001	19.176	< 0.001	6.431	< 0.001
	roots	20.531	< 0.001	13.742	< 0.001	10.701	< 0.001
Fructose	leaves	71.449	< 0.001	31.287	< 0.001	6.021	< 0.001
	twigs	3.613	0.046	20.447	< 0.001	2.119	0.033
	stems	4.724	0.021	21.152	< 0.001	5.840	< 0.001
	roots	48.172	< 0.001	31.263	< 0.001	23.429	< 0.001
Sucrose	leaves	25.039	< 0.001	53.144	< 0.001	14.157	< 0.001
	twigs	1.061	0.402	60.225	< 0.001	4.309	0.005
	stems	1.660	0.228	24.773	< 0.001	2.502	0.012
	roots	14.311	< 0.001	135.578	< 0.001	11.022	< 0.001
Soluble sugar	leaves	21.218	< 0.001	9.923	< 0.001	9.435	< 0.001
	twigs	2.707	0.092	13.054	< 0.001	1.318	0.285
	stems	5.478	0.013	14.219	< 0.001	2.260	0.023
	roots	6.572	0.007	50.562	< 0.001	4.659	0.001
Starch	leaves	14.041	< 0.001	15.117	< 0.001	5.623	0.001
	twigs	16.603	< 0.001	35.201	< 0.001	5.163	< 0.001
	stems	15.254	< 0.001	15.452	< 0.001	1.828	0.143
	roots	81.858	< 0.001	12.909	< 0.001	13.511	< 0.001
Total NSC	leaves	28.888	< 0.001	13.033	< 0.001	10.755	< 0.001
	twigs	12.253	0.001	14.215	< 0.001	3.282	0.002
	stems	9.994	0.001	10.799	< 0.001	1.816	0.125
	roots	15.978	< 0.001	36.825	< 0.001	18.944	< 0.001
Soluble sugar: starch	leaves	5.453	0.013	12.374	< 0.001	3.490	0.008
	twigs	17.240	< 0.001	31.270	< 0.001	3.443	0.022
	stems	26.882	< 0.001	28.115	< 0.001	3.452	0.001
	roots	39.121	< 0.001	19.478	< 0.001	8.562	0.001
